# A multi-phase deep CNN based mitosis detection framework for breast cancer histopathological images

**DOI:** 10.1038/s41598-021-85652-1

**Published:** 2021-03-18

**Authors:** Anabia Sohail, Asifullah Khan, Noorul Wahab, Aneela Zameer, Saranjam Khan

**Affiliations:** 1 Pattern Recognition Lab, DCIS, Pakistan Institute of Engineering and Applied Sciences (PIEAS), Nilore, Islamabad, 45650 Pakistan; 2 Deep Learning Lab, Centre for Mathematical Sciences, Pakistan Institute of Engineering and Applied Sciences, (PIEAS), Nilore, Islamabad, 45650 Pakistan; 3 Department of Computer Science, Tissue Image Analytics (TIA) Lab, University of Warwick, Coventry, UK; 4 Department of Physics, Islamia College Peshawar, Peshawar, Pakistan

**Keywords:** Cancer, Computational biology and bioinformatics

## Abstract

The mitotic activity index is a key prognostic measure in tumour grading. Microscopy based detection of mitotic nuclei is a significant overhead and necessitates automation. This work proposes deep CNN based multi-phase mitosis detection framework “MP-MitDet” for mitotic nuclei identification in breast cancer histopathological images. The workflow constitutes: (1) label-refiner, (2) tissue-level mitotic region selection, (3) blob analysis, and (4) cell-level refinement. We developed an automatic label-refiner to represent weak labels with semi-sematic information for training of deep CNNs. A deep instance-based detection and segmentation model is used to explore probable mitotic regions on tissue patches. More probable regions are screened based on blob area and then analysed at cell-level by developing a custom CNN classifier “MitosRes-CNN” to filter false mitoses. The performance of the proposed “MitosRes-CNN” is compared with the state-of-the-art CNNs that are adapted to cell-level discrimination through cross-domain transfer learning and by adding task-specific layers. The performance of the proposed framework shows good discrimination ability in terms of F-score (0.75), recall (0.76), precision (0.71) and area under the precision-recall curve (0.78) on challenging TUPAC16 dataset. Promising results suggest good generalization of the proposed framework that can learn characteristic features from heterogenous mitotic nuclei.

## Introduction

Breast cancer is the second most commonly diagnosed cancer, which has affected women’s lives globally^1^. It most commonly occurs due to irregular cell division of breast tissue such as milk duct or lobules, resulting in the formation of tumour. Mitotic activity index (number of dividing cells under 2 mm^2^ tissue area) is the key factor for determining tumour size, proliferation rate, and aggressiveness^2^. Due to the imperative importance of mitosis count, it is considered as a prognostic measure in Bloom-Richardson’s grading system^3^. In a routine histopathology workflow, a pathologist analyses the biopsy sample of the affected region under a microscope and counts the number of mitotic nuclei in 10 High Power Field (HPF) areas. However, manual analysis is tiresome and time-consuming as there are, low density of mitotic nuclei per HPF surrounded by an overwhelming number of other cellular components^4^. Moreover, it is highly dependent upon the experience of a pathologist and may suffer from inter- and intra-observer variability due to the element of human subjectivity^5^. The development of an automated mitosis detection system is thus required to reduce the burden on pathologists and to improve the objectivity of their decisions by providing an additional opinion.

With the advent of digital pathology, many computational algorithms have been developed for the automation of pathological workflow. Recent advances in deep convolutional neural networks (CNNs) and their commendable performance on image classification, detection, and segmentation have accelerated their use in medical imaging problems^6–8^. Deep CNNs are a type of representative learning algorithms that automatically extract the relevant information from raw images without putting effort into manual designing of feature descriptors^9^. CNN based models have been successfully applied in several histopathology problems with success, for instance: categorization of breast tissue^10^ into normal, benign, in situ and invasive carcinoma, detection of cancer metastasis^11^, quantification of lymphocytes^12^, demarcation of tumour region^13^, segmentation of cell nuclei and many others^14^.

**Figure 1:**
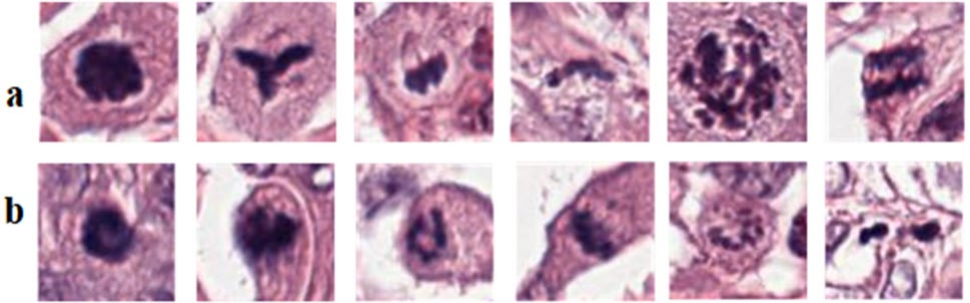
Histopathology patches from TUPAC16 dataset showing heterogeneity in mitosis appearance and their close similarity with non-mitosis, panels (**a**, **b**) showing mitotic and non-mitotic nuclei, respectively.

Similarly, several CNN based approaches have been proposed to detect mitosis; however, these algorithms still have a margin of improvement due to the challenging nature of the problem^15–17^. Automated detection of mitotic nuclei is challenging due to their atypical configuration and difference in the texture of cells in different morphological phases (shown in Fig. 1). Mitotic cell division is characterized by four different stages: prophase, metaphase, anaphase, and telophase^18^. For instance, telophase is distinct, as, in this phase, the nucleus is divided into two distinct parts and is still considered as a single cell. Besides, mitotic nuclei resemble many other hyperchromatic cellular bodies such as necrotic, apoptotic and non-dividing dense nuclei, making detection of mitosis difficult on tissue patch^19^ (shown in Fig. 1). Moreover, tissue slides vary in appearance due to acquisition from different pathology labs and their preparation protocols.

**Figure 2:**
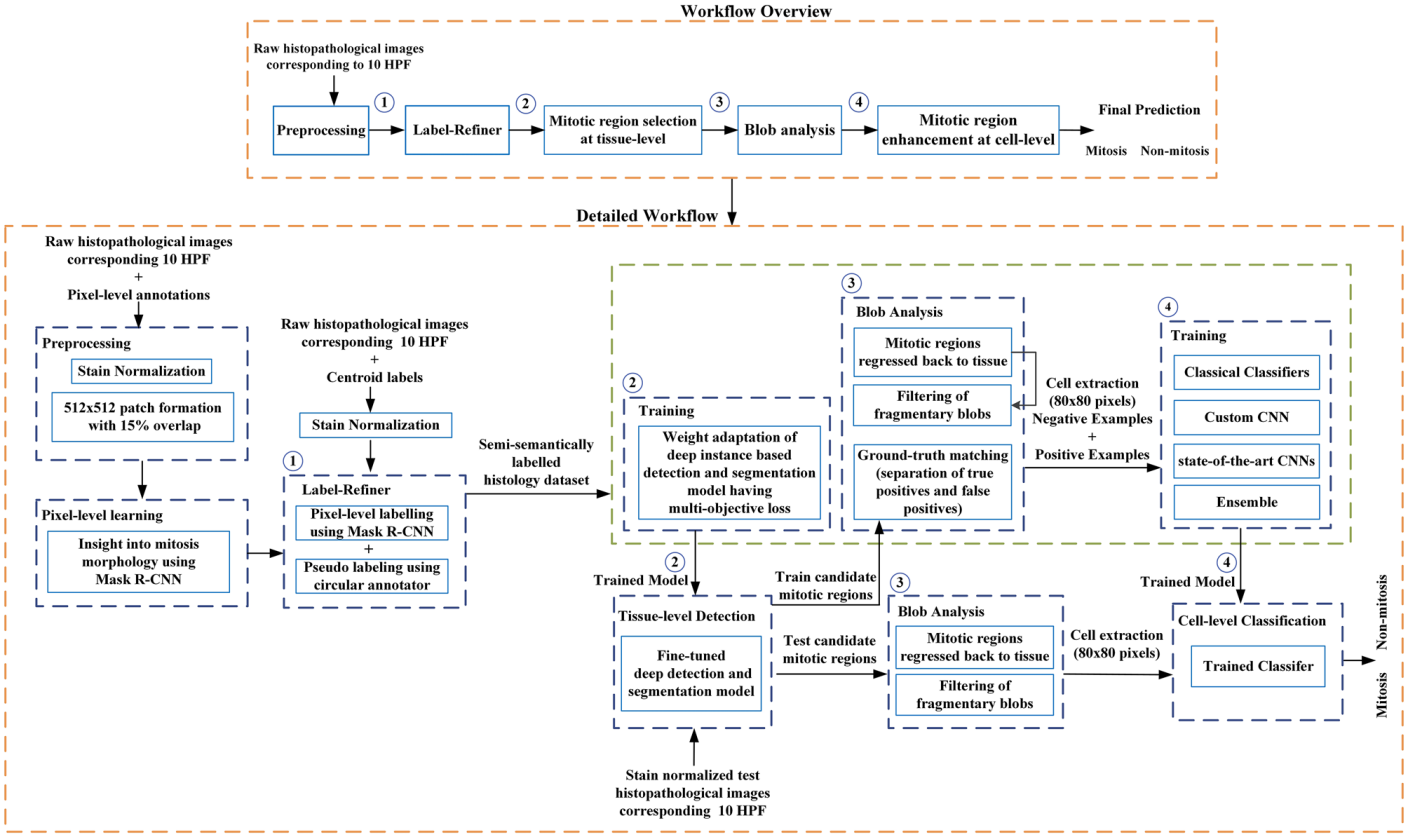
Complete workflow of the proposed multi-phase mitosis detection framework (MP-MitDet).

To address all of the above mentioned challenges, in this study we have proposed a new learning framework for weakly labelled dataset. In this regard, a deep CNN based multi-phase mitosis detection framework “MP-MitDet” is developed that analyses the Haematoxylin and Eosin (H&E) stained breast cancer histopathology images (work flow is shown in Fig. 2). Initially, a weakly labelled mitosis dataset, with centroid labels only, is refined by adding pixel-level semantic information. The resulting dataset with refined labels is assigned to a deep detection module for mitotic nuclei identification. A deep instance-based detection and segmentation CNN is employed on tissue level to localize the probable mitotic regions, thus neglecting numerous non-mitotic nuclei. Blob analysis is performed on the selected region to filter fragmentary probable mitotic nuclei. Enhancement of the preceding phase is performed on cell level via a proposed deep custom “MitosRes-CNN” to remove the false-mitoses. Different augmentation and colour variation strategies are applied during training to address the class imbalance problem and to make the classifier robust towards unseen patient examples. The contributions of the proposed framework are the following:

(i) Exploitation of deep segmentation model using transfer learning (TL) to improve the labels of weakly annotated mitoses by learning morphological information from small patient dataset.
(ii) Exploitation of instance-based detection and segmentation model using multi-objective loss to reduce the class imbalance with minimum loss of mitosis.
(iii) Concept of weight transfer and cross-domain TL are exploited to adapt the deep architectures on small dataset.
(iv) Custom-made deep CNN “MitosRes-CNN” is proposed that is robust against the mitotic appearance variability.
(v) Comparative analysis of the proposed “MitosRes-CNN” is provided with diverse categories of state-of-the-art CNN architectures for mitosis detection problem.

## Related work

Several competitions held by ICPR12, AMIDA13, ICPR14, and TUPAC16 have provided the benchmarked dataset for the automation of mitosis detection problem^19–22^. The proposed detection approaches can be broadly categorized into classical and CNN based data-driven approaches. Classical approaches reported in literature often exploit quantitative features, for instance, morphology, colour, texture and statistical features to represent the mitosis and assign these representations to the classifier for discrimination^23,24^. Contrary to classical approaches, CNN based approaches are superior in performance as they eliminate the reliance on the handcrafted features. Currently, exiting CNN based approaches can be categorized as pixel-level classification or fine-grained detection approaches where initially candidate mitotic regions are identified that are assigned to another classifier to improve the detection.

There are a few techniques that have used end-to-end pixel-wise classification scheme for mitosis detection. In earlier studies, Ciresan et al. (2013) developed a max-pooling based Fully Convolutional Net (FCN) that performed pixel-wise classification. The developed model was applied to the unseen data in a sliding window manner to locate the mitoses^25^. The proposed approach stood at the top place in the ICPR12 challenge with an F-score of 0.78. However, this approach is slow and computationally intensive. Zerhouni et al. (2017) proposed use of state-of-the-art Wide Residual Networks for pixel-wise classification of mitosis on breast histology images. Post-processing was applied to the output to remove the noise and less probable mitoses, whereas the final decision was taken by combining the output of several networks using the majority vote^26^.

Due to the intricate nature of mitosis detection problem, most of the techniques address it by employing multiple detection and classification models. ICPR14 dataset, instead of providing the morphological information of the mitosis, only provided centroid annotation, thus increasing the difficulty level of detection. Chen et al. (2016) overcame the challenging nature of the dataset by adopting a deep cascaded CNN based approach and this technique won the ICPR14 challenge^27^. In the first phase of Chen et al.’s technique, an FCN was used to locate the candidate mitoses. In contrast, in the second phase, a fine discrimination model was developed that utilized the cross-domain knowledge to remove false positives. Wahab et al. (2017) proposed a two-phase classification approach to deal with the class imbalance problem. Initially, the dataset for classification was created by performing global binary thresholding on blue ratio images^28^. In the first stage of the proposed technique, CNN was trained on the original dataset to identify the hard examples. Whereas in the second stage, the dataset was improved by performing the under-sampling of the negative examples using histogram-based k-means clustering on blue ratio images and augmented the dataset by including hard negative examples. In another study, Wahab et al. (2019) proposed TL based efficient mitosis classifier^29^. They initially used customized pre-trained FCN for the mitosis detection, whereas in the second phase, predictions of the first phase were refined by assigning the output to another CNN that was hybrid of AlexNet and custom layers. The mitosis detection technique that got first place in the TUPAC16 challenge also adopted a two-stage detection approach to get an improved F-score^30^.

Region based CNNs (R-CNNs)^31^ have shown good performance for object detection problems in computer vision. Therefore, Li et al. (2018) exploited the region information of the mitosis using VGG16 backboned faster R-CNN to filter out the probable mitotic regions that are further refined by assigning the predictions to another deep network to remove false positives^32^. Likewise, MitosisNet proposed by Alom et al. (2020) also consisted of multiple deep learning models, including segmentation, detection, and classification models for the final decision of the mitosis regions^33^. Similarly, Mehmood et al. handle the complex nature of mitosis by initially identifying probable mitotic regions through R-CNN. They isolated non-mitotic regions from the selected regions by assigning the initial phase results to the ensemble of ResNet50^34^ and DenseNet201^35^. The proposed approach achieved 0.858 and 0.691 F-score for the ICPR 2012 and ICPR 2014 dataset, respectively.

Mitosis labelling is tedious, and it is not easy to rigorously annotate them on the Whole Slide Images (WSIs). To overcome this limitation, Akram et al., proposed a semi-supervised learning model for WSIs that shows promising results on the TUPAC16 dataset^36^. Initially, the detection model was trained on a small size labelled dataset. This trained model was further used to filter out the mitosis samples from unlabelled WSIs. In this way, a large dataset was built, used for the final training of the detection model.

Training of a deep learning model with a weakly annotated dataset is challenging. This problem is addressed by Li et al. (2019), who introduced a new learning scheme to identify the mitotic nuclei using semantic segmentation based FCN. Their approach achieved state-of-the-art results on the MITOS14 dataset, AMIDA13 dataset, and TUPAC16 dataset with an F-score of 0.562, 0.673, and 0.669, respectively^15^. The defined scheme uses concentric circular labels to represent the mitotic region and proposed a concentric loss function that only considers the region inside the circle whereby it excludes the chance of non-mitotic region overlap with the mitotic region. Similarly, Sebai et al. (2020) adapted the semantic segmentation model for the mitosis detection problem^16,54,53^. They handled the issue of weak labels by integrating two deep networks in an end to end manner. They separately trained both networks on weak and pixel-level labels in a simultaneous way. The final decision was made by combining the prediction of both the models. Most of the techniques discussed above are based on state-of-the-art pre-trained CNN models without custom modifications that were previously reported in literature.

## Methods

This work suggests a multi-phase deep CNN based mitosis detection framework (MP-MitDet) for H&E stained breast cancer histopathological images. The workflow is decomposed into 4 phases: (1) refinement of weakly labelled mitosis dataset, (2) mitotic region selection at tissue-level, (3) blob analysis, and (4) enhancement of mitosis detection results at cell-level. The overall workflow of the proposed detection model is shown in Fig. 2.

### Datasets

TUPAC16 challenge provided a publicly accessible dataset for mitosis detection problem named TUPAC16 auxiliary dataset^21^. This dataset consisted of specified regions of breast biopsies corresponding to 10 HPF selected by the pathologists from WSIs. The provided images were collected from three different centres of Netherlands and were scanned by two different scanners under 40 × magnification. The dataset consisted of 656 images collected from 73 patients. This challenge provided a maximum number of patient samples to date for mitosis detection problem, whereby it provided 50 new patients and included 23 patients’ samples from the AMIDA13 challenge. This dataset is challenging as it provided only centroid labels of the mitoses. Provided mitoses were annotated rigorously by two pathologists. In addition to the TUPAC16 dataset, patient samples from two other previous challenges: MITOS12 and MITOS14 were also included in the training to augment the small number of patient samples and to improve the learning of deep CNN models (details of the dataset are mentioned in Table 1)^20,22^. All three different datasets were H&E stained and taken from breast tissue biopsies.

**Table 1:** Details of the datasets.

Dataset	Scanner	Resolution (μm/pixel)	Spatial Dimension	Patients	Mitosis
TUPAC16	Aperio ScanScope	0.25	2000 × 2000	23	914
	Leica SCN400	0.25	5657 × 5657	50	
MITOS12	Leica SCN400	0.2456	2084 × 2084	5	226
	Hamamatsu	0.2275	2252 × 2250	5	
MITOS14	Leica SCN400	0.2456	1539 × 1376	11	749
	Hamamatsu	0.2275	1663 × 1485	11	

### Cross-validation scheme

The dataset was divided into train, validation, and test sets, whereas the patient samples in the test were kept the same as mentioned in Wahab et al.’s (2019)^37^ study to make the results comparable. Data was divided in such a way that the patient samples were kept disjointed in train, validation, and test to emulate the real-world scenario. The data division is mentioned in Table 2. Cross validation scheme is kept the same for deep instance based detection and segmentation model employed for tissue level detection as well as for classical and deep classification models used for cell-level classification. All the models were trained with the training dataset and hyperparameters and architectural configurations were selected based on model performance on validation dataset. However, test dataset was kept separate from the training and validation set, and it was used for the final evaluation of the selected models.

**Table 2:** Cross-validation scheme for detection and classification models.

Dataset	Patient Number
Train	TUPAC16 patients: 01, 02, 03, 05, 07, 08, 10, 11, 12, 13, 14, 15, 16, 17, 18, 19, 20, 22, 23, 24, 25, 28, 33, 34, 35, 37, 38, 40, 42, 44, 47, 49, 51, 52, 54, 59, 61, 64, 68, 69, 70, 72 All patients from training dataset of MITOS12 and MITOS14
Validation	04, 06, 09, 21, 26, 29, 31, 39, 46, 48, 56, 65, 67, 73
Test	27, 30, 32, 36, 41, 43, 45, 50, 53, 55, 57, 58, 60, 62, 63, 66, 71

### Preprocessing and normalization of images

**Figure 3:**
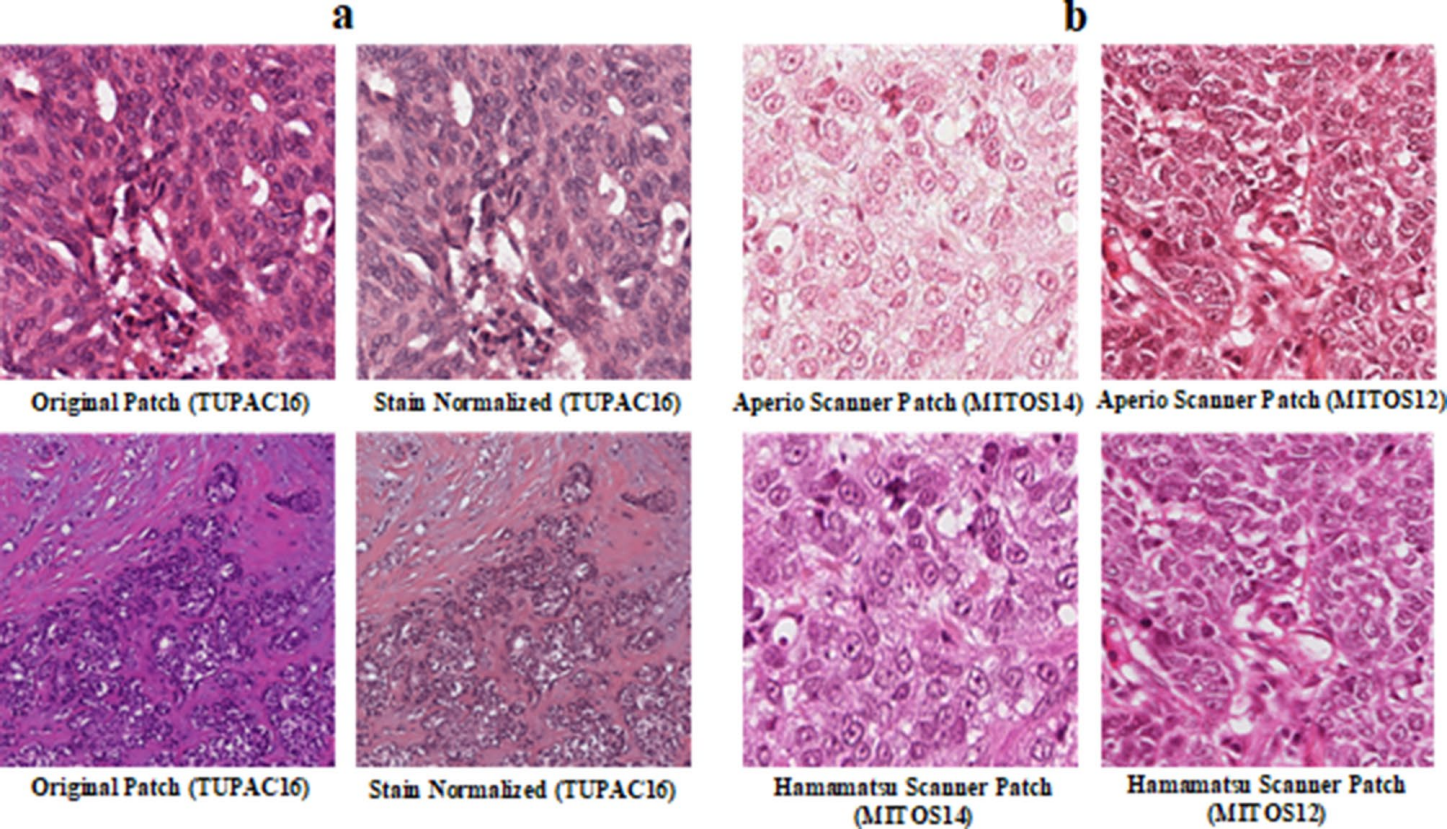
(**a**) Actual vs. stain normalized images; (**b**) histopathological images from different scanners.

Histopathological images were stain normalized initially using Macenko et al.’s^38^ technique to mitigate the variations in staining colours. It is expressed in Eq. (1–3). Histopathological images were collected from different pathological laboratories. Each laboratory has its staining protocols, and samples were digitalized using the different scanner, resulting in noise and non-uniformity in colour appearance (shown in Fig. 3). All the images were mean normalized and standardized (Eq. 4) before assigning them to the machine learning models.

1 $$OD\, = \, - \log_{10} (H)$$

2 $$OD\, = \,VS$$

3 $$S = V^{ - 1} OD$$

4 $$H_{norm} = \frac{{H_{snorm} - \mu }}{\sigma }$$

In Eq. (1), $H$ represents histopathological image whose RGB values are transformed to optical density values $(OD)$. Whereas, in Eq. (2), $V$ represents the stain vectors for H&E, and $S$ shows their saturation value. Equation (3) is used to express the stain values in a standard quantitative way. In Eq. (4), $H_{snorm}$ and $H_{norm}$ are the stain normalized input and mean-standard deviation normalized pixel values, respectively, whereas $\mu$ and $\sigma$ are used to represent the mean and standard deviation for RGB values of the dataset.

### Refinement of weakly labelled mitosis dataset

**Figure 4:**
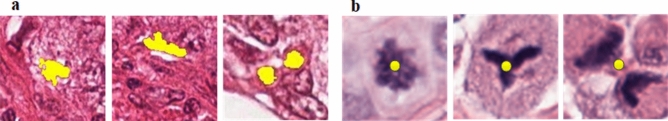
(**a**) Pixel-level annotations; (**b**) weak labels (centroid labels).

**Figure 5:**
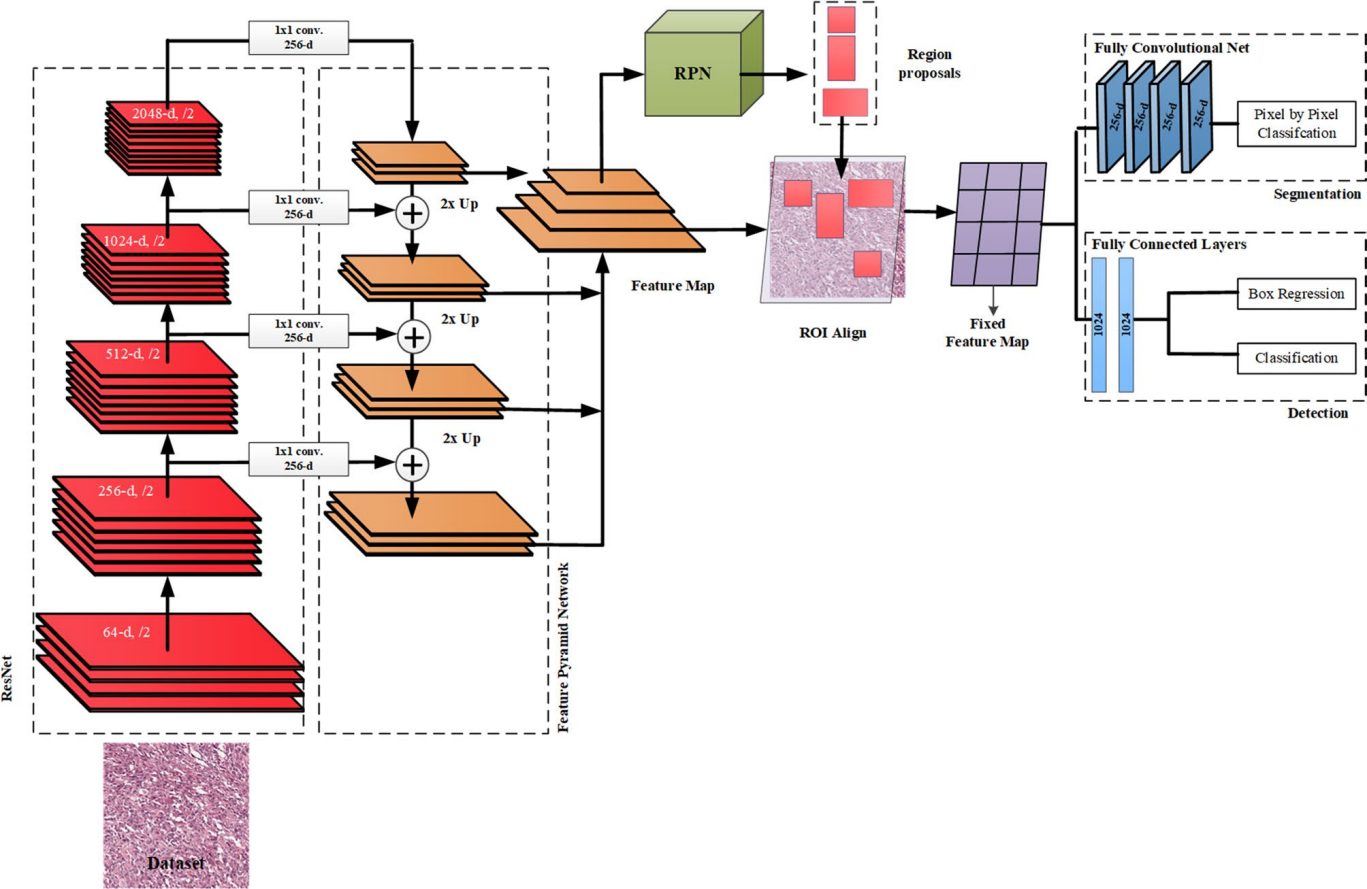
Architectural details of the Mask R-CNN.

Annotations for the MITOS14 and TUPAC16 datasets are provided as weak labels $(Y_{centroid} = M_{x,y = c} )$. The labels are represented by centroid pixel ($M_{x,y = c}$) of the mitoses, as shown in Fig. 4b. In practice, it is extremely time-consuming for pathologists to annotate a large number of patient samples manually and assign pixel-level labels. Therefore, an automated labelling approach named as label-refiner is developed to assign the pixel-level labels. Mask R-CNN^39^ is used to develop the fine masks for mitoses and it is trained with MITOS12 dataset (Fig. 4a) having pixel-level annotations $(Y_{polygon} = M_{x,y = 1}^{s} )$. MITOS12 dataset consists of only 5 patients (Table 1), therefore pretrained Mask R-CNN is used and fine-tuned on 338 samples from 5 patients. Strongly labelled dataset helps the Mask R-CNN to learn the morphology of the mitoses. Mask R-CNN architecture is shown in Fig. 5. The learning stages of Mask R-CNN are divided into (i) Feature Extraction Network (ResNet + Feature Pyramid Network), (ii) Region Proposal Network (RPN), (iii) Region of Interest (ROI) alignment, (iv) Detection and Segmentation.

**Figure 6:**
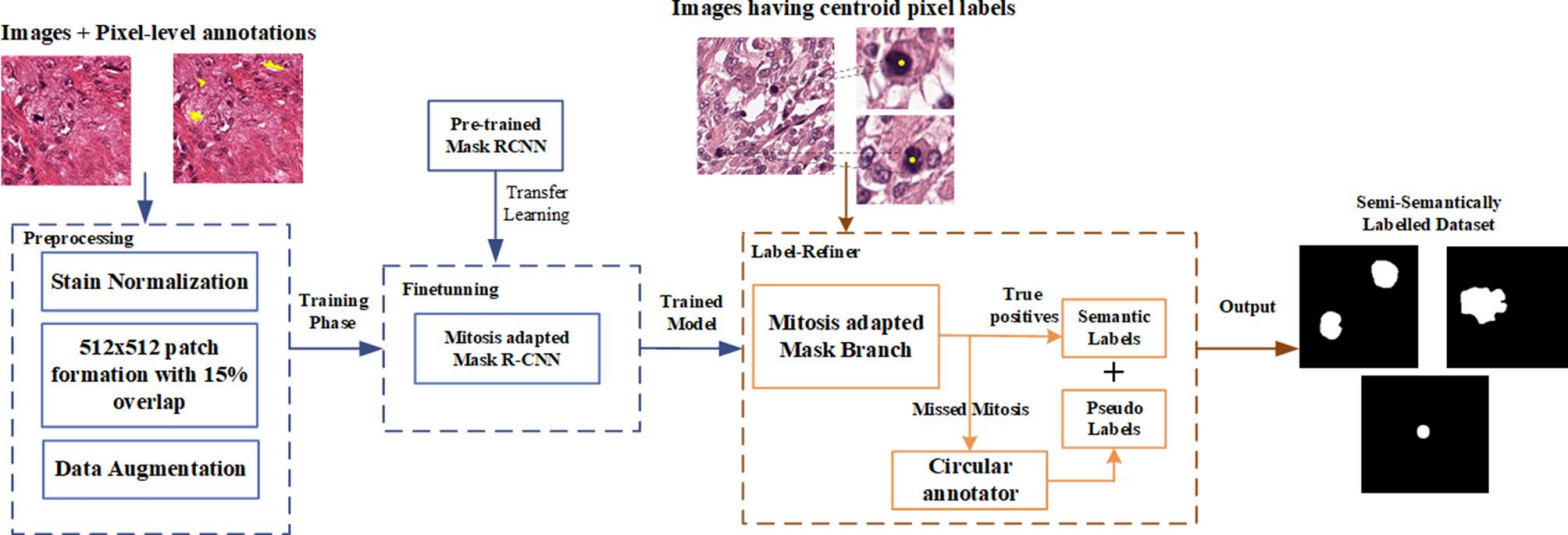
Label refinement module (label-refiner) for the weakly annotated dataset.

The trained Mask R-CNN is used to generate the pixel by pixel masks for the training set (Table 2) of mitosis detection module. Generated masks are used as labels for further analysis of the mitosis dataset, whereas segmented blobs that do not correspond to true mitoses are discarded. During the refinement phase, some labels are missed by the detection model for which pseudo labels $(\,Y_{circular} )$ are developed. Pseudo labels $(\,Y_{circular} )$ are generated based on the idea proposed by Li et al. (2019)^15^ and represented each mitosis in the form of a circle. Circular annotation is drawn randomly with a radius of 10–16 pixels. So, the developed label-refiner $f_{refiner} (H_{norm} ,\,Y_{centroid} )$ (expressed in Eq. (5)) works by taking input images $(H_{norm} )$ and their weak labels $(\,Y_{centroid} )$ and returns the semi-semantically labelled dataset $(Y_{semi - semantic} )$, consisting of true morphology and pseudo representation. The complete workflow of the label refinement phase is shown in Fig. 6.

5 $$Y_{semi - semantic} = f_{refiner} (H_{norm} ,\,Y_{centroid} )$$

### Mitotic region selection at tissue level

**Figure 7:**
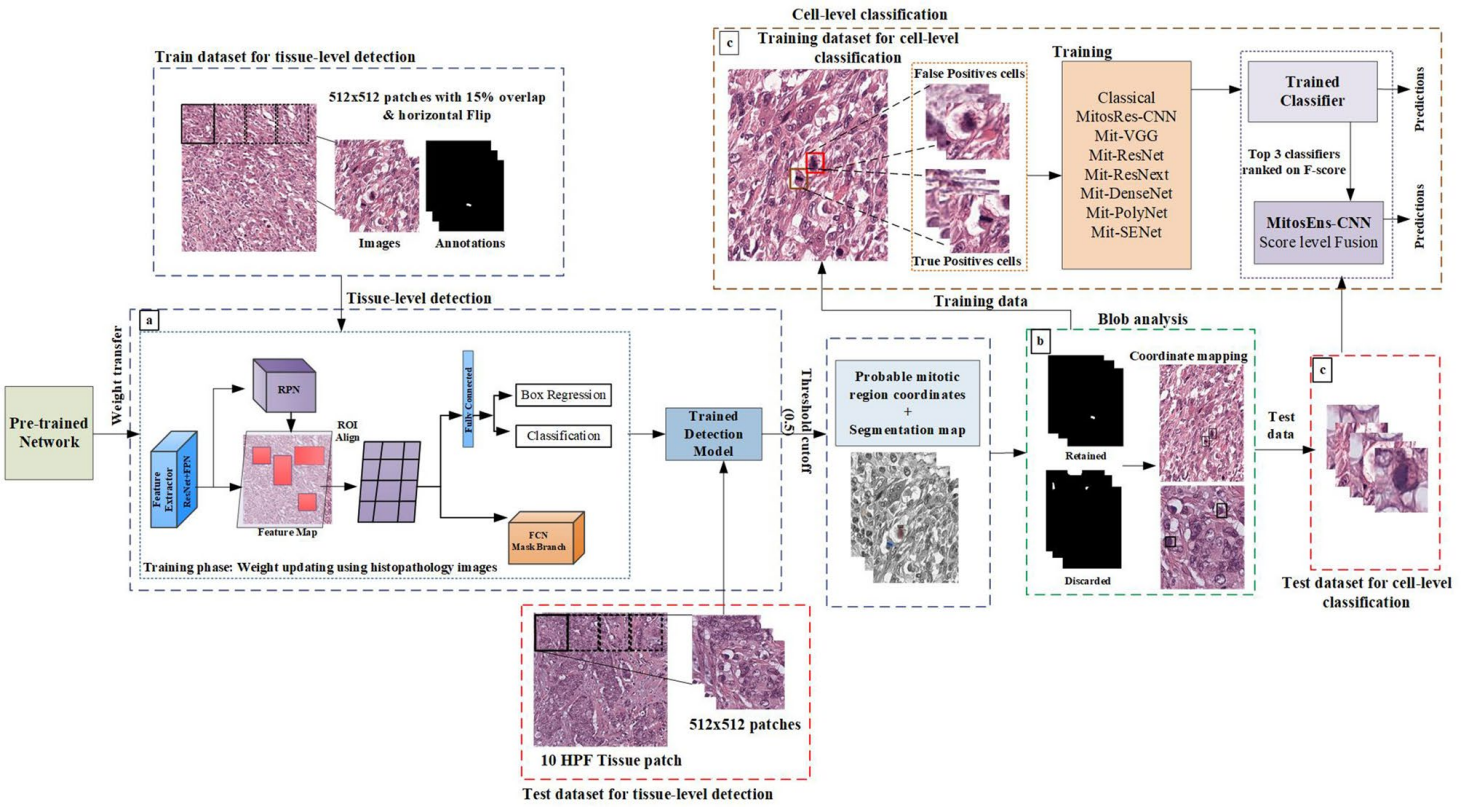
Work flow diagram, (**a**) mitotic region selection at tissue-level using multi-objective deep instance based detection and segmentation model, (**b**) blob analysis, (**c**), enhancement using cell-level classification.

We exploited multi-objective loss function of Mask R-CNN for mitosis region selection from histopathology tissue patches using weight space TL and hyper-parameter optimization. Workflow of mitotic region selection at tissue-level using multi-objective deep instance based detection and segmentation model is shown Fig. 7a. The use of multi-objective loss function ($L_{multi - obj}$) expressed in Eq. (6) helps to improve the mitosis detection task by incorporating the morphology information (Eq. (9)) from segmentation branch along with region information from detection branch that performs bounding box regression and classification (Eq. (7 & 8)).

6 $$L_{multi - obj} = l_{clas} + l_{reg} + l_{mask}$$

7 $$l_{clas} = - \log P(C_{j}^{*} |C_{j} )$$

8 $$l_{reg} = smooth\,L_{1} (B_{j} - B_{j}^{*} )$$

9 $$l_{mask} (C^{*} ,C) = \frac{1}{R \times H}\sum\limits_{r = 1}^{R} {\sum\limits_{c = 1}^{C} {\left[ {C_{r,c}^{*} .\log C_{r,c} + (1 - C_{r,c}^{*} ).\log (1 - C_{r,c} )} \right]} }$$

Equation (7 & 8) represent the classification $(l_{clas} )$, and box regression $(l_{reg} )$ loss, respectively, whereas $C_{j}$ and $B_{j}$ denote the predicted class label and estimated bounding box coordinates, respectively, for *j*^*th*^ anchor. Likewise, $C_{j}^{*}$ and $B_{j}^{*}$ are used to represent the ground-truth values for *j*^*th*^ anchor. Bounding box regression branch returns the real values therefore the difference between ground-truth spatial coordinates and predicted coordinates is minimized by computing smooth L1 loss (Eq. (8)). The segmentation loss function $(l_{mask} )$ is estimated by using Eq. (9), whereas spatial dimensions of the input region proposal are represented via $(R \times H)$. In this equation, $(.)$ operator denotes the multiplication sign, whereas $C_{r,c}^{*}$ indicates the ground-truth class for $(r,c)$ coordinates and $C_{r,c}$ shows predicted class probability at $(r,c)$ coordinates of the image.

In this study, mitotic region selection is considered as a binary class detection problem. For the training of detection module (Mask R-CNN), histopathological images are labelled in a COCO format^40^ by defining mitosis as an object of interest, whereas all other cellular/nuclear components and stroma are labelled as background. The backbone of Mask R-CNN was built on Feature Pyramid Network based ResNet101^34,41^ shown in Figs. 5 and 7 for the learning of enriched deep feature hierarchies. The advantage of using this network is that it learns strong semantic information while retaining spatial information at different scales. Mitoses are small in size and are expressed via few pixels in the later layers of deep architecture however, this information is not enough for object detection. Feature Pyramid Network (FPN) alleviates this problem by extracting the feature representation of the object at different scales^41^. Feature representation learned from FPN is assigned to the RPN to extract the probable regions. In RPN, region proposals are searched by using 12 anchors by setting aspect ratios as {1:2, 1:1, 2:1} on four different scales {32 × 32, 64 × 64, 128 × 128, 256 × 256}. RPN selects the region proposals by computing Intersection over Union (IoU) of each region proposal with ground truth and retains only those regions that overlap by at least 70%. These region proposals are mapped to the feature map using ROI align that resizes them using bi-linear interpolation and assigns them to the detection and segmentation head for the classification of the selected regions.

### Training of detection model using weight space transfer learning

Mask R-CNN is trained on 3904 images of train dataset (Table 2) by exploiting the idea of weight transfer of pre-trained architecture to adapt the deep architecture on a small mitosis dataset consisting of 61 patients^42^. Deep NNs usually require a massive amount of data for training and suffer from overfitting on insufficient data. TL addresses this problem by allowing to reuse the knowledge of the pre-trained network to a new task and has shown remarkable results when labelled data is insufficient in the target domain. TL can be employed by using pre-trained architecture as a fixed feature extractor, freezing lower layers and fine-tuning higher layers, or leveraging parameter space of pre-trained architecture to the target domain^43^.

In this work, the backbone architecture of Mask R-CNN is pre-trained on 1 million natural images from ImageNet, whereas the detection and segmentation head is pre-trained on the COCO dataset^40,44^. So, we define the source domain as $D(S) = \left\{ {I_{N} ,P(I_{N} )} \right\}$ where $I_{N}$ represents the natural images and $P(I_{N} )$ represents its marginal distribution. The knowledge domain of a pre-trained network $N_{S} = f_{S} (\theta_{S} ,\left\{ {I_{N} ,Y_{N} } \right\})$ constitutes the images $(I_{N} )$, labels $(Y_{N} )$, and parameter space $(\theta_{S} )$. During training, we adapted the parameter space $(\theta_{S} )$ by finetuning the network end to end by assigning histopathological images $(I_{H} )$ from the target domain $D(T) = \left\{ {I_{H} ,P(I_{H} )} \right\}$. This finetuned architecture is defined by $N_{T} = f_{T} (\theta_{T} ,\left\{ {I_{H} ,Y_{H} } \right\})$ and is used for mitotic region selection.

### Blob analysis

In this phase, Mask R-CNN predictions are filtered out at threshold cut-off of 0.5 and all regions that are predicted with more than 50% confidence as mitotic regions are regressed to patient’s tissue patches (Fig. 7b). Candidate mitotic nuclei that are selected are considered as blobs. On the selected regions, blob analysis is performed to retain blobs with an area more than 600 pixels and fragmentary blobs are removed. Bounding boxes for training dataset are analysed and the false positives are separated from the true positives based on their centroid distance from ground truth (Fig. 7c). Predicted bounding boxes that do not lie within the 30-pixel distance of ground truth are considered as false positives, whereas other predictions are kept as true mitosis.

### Enhancement of mitosis detection results at cell-level

In the last phase, refinement of the selected mitotic regions is performed to reduce the false positives (Fig. 7c). Therefore, custom made deep CNN “MitosRes-CNN” is proposed for the analysis of selected cells to discriminate hard negative examples from true mitoses. Network topology including depth and width are selected based on validation dataset. The performance of the proposed architecture is compared with the state-of-the-art deep CNN architectures and classical machine learning models. Details of this section are mentioned below.

### Augmentation and oversampling of the mitotic nuclei

Histopathological images exhibit colour appearance multiplicity due to variation in staining protocols across labs and image acquisition under different scanners. Different image variations are applied on-the-fly during the classifier’s training to make it robust towards unseen patient examples. Augmentation strategies include horizontal and vertical flip, rotation, and colour jitters. Mitotic examples are augmented by extracting patches at various positions to avoid the overfitting. Furthermore, during training, the effect of imbalance is reduced by controlling the proportion of minority and majority class by fetching more positive examples based on the ratio of negative and positive examples. Validation and test sets are drawn from patients without augmentation to emulate the real-world scenarios.

#### Proposed MitosRes-CNN for mitosis classification

**Figure 8:**
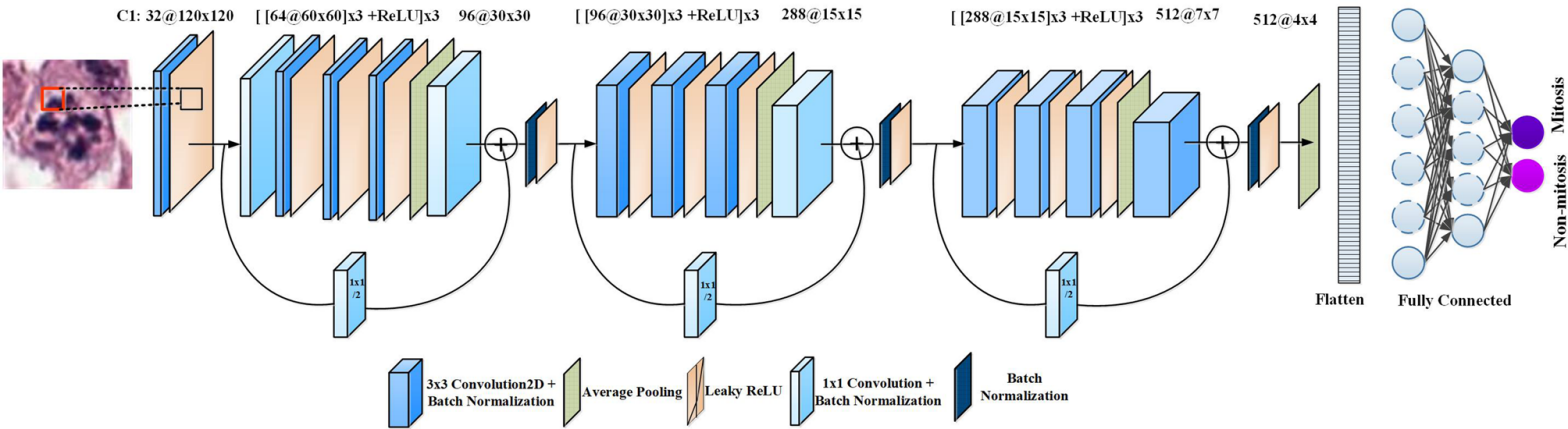
Block diagram of the proposed MitosRes-CNN.

In this work, we proposed a new custom CNN “MitosRes-CNN” for the discrimination of false positives from the true mitotic nuclei. The proposed architectural scheme is shown in Fig. 8. The proposed MitosRes-CNN is formulated of three custom residual blocks with shortcut links. Residual learning^34^ [Eq. (10–12)] is implemented to tackle the problem of vanishing gradient. Moreover, it performs reference-based optimization of weights by comparing transformed feature-maps with input feature-maps, [as shown in Eq. (10–12)] thus it encourages each block to learn useful features.

Within each custom residual block, set of transformations are implemented to better approximate the data representation, whereas the concept of the effective receptive field^45^ is exploited by convolving the feature-maps with the same size of filters. The transformations are comprised of 3 × 3 convolution operation (Eq. (10)) in combination with batch normalization to smoothen the gradient-based learning^46^. Leaky ReLU (Eq. (12)) is used as a activation function to incorporate the non-linearity and to add the sparsity^47^. Leaky ReLU addresses the problem of dead neurons by permitting the positive activation ($a\,$) values as such, whereby assigning a small gradient to negative activations ($a\,$).

10 $$O_{x,y}^{l + 1} = \sum\limits_{c}^{C} {\sum\limits_{p,q}^{P,Q} {k_{i,j} \,M_{p + i,q + j}^{l} } } + b$$

11 $$M_{x,y}^{l + 1} = O_{x,y}^{l + 1} + M_{x,y}^{l}$$

12 $$M_{x,y}^{l} = M_{x,y}^{l + 1} - O_{x,y}^{l + 1}$$

13 $$f(a) = \left\{ {\begin{array}{*{20}l} a \hfill & {if\,a\, > \,0} \hfill \\ {0.01a} \hfill & {otherwise} \hfill \\ \end{array} } \right\}$$

Equation (10) expresses convolution operation performed by convolution layer, whereas $M_{p,q}^{l}$ represents the input feature map of spatial size $(P \times Q\,{\text{pixels}})\,$ for $l{\text{th}}$ layer, $k$ denotes the kth convolution kernel and b is for bias. Whereas, *c* shows the feature map depth, (*i, j*) represents spatial coordinates of kernel and (*p* + *i, q* + *j*) shows the receptive field occupied by kernel. In Eq. (11), $M_{x,y}^{l}$ is the input assigned to residual block, whereas $O_{x,y}^{l + 1}$ is a transformed (convolved) feature-map that is output from $l{\text{th}}$ layer. Equation (12) shows the residual output ($M_{x,y}^{l + 1} - O_{x,y}^{l + 1}$).

14 $$A_{x,y}^{l + 1} = f_{avg} (M_{x,y}^{l + 1} )$$

At the end of each residual block, downsampling of the feature-map is performed using average pooling (Eq. (14)) to incorporate both low and high-level responses. In Eq. (14) average pooling operation is denoted via $f_{avg} (.)$ and $A_{x,y}^{l + 1}$ represents its outputs for *l*^*th*^ layer. Pooling operation helps in reducing the overfitting and to learn the invariant features. Average pooling operation is followed by a 1 × 1 convolution to increase the number of feature-maps^9^. The number of feature-maps is increased three times at the end of each block. Each block assigns its output to the next block in a feed-forward manner as well as connected via shortcut link (Eq. (11 & 12)) to provide direct access to the gradient.

Dropout with a 50% probability is used for fully connected layers to limit the overfitting. SoftMax is used at the end of a fully connected layer to compute the probability of each sample belonging to mitosis or non-mitosis. Details of the proposed architecture are mentioned in Table 3. Weights (W) of the proposed network are initialized using Glorot uniform random weight initialization strategy (Eq. (15)) and bias (b) is initialized with 0.

15 $$W\sim U\left[ { - \frac{1}{z},\frac{1}{z}} \right],\,\,U\left[ { - n,n} \right]$$

**Table 3:** Architectural details of the proposed MitosRes-CNN.

Layer number	Processing unit	Input	Output	Filters	Filter size	Stride	Zero padding
Input layer	Conv2D + BatchNorm2D + Leaky ReLU	3 × 120 × 120	32 × 60 × 60	32	3 × 3	2	1
Block 1	Conv2D + BatchNorm2D	32 × 60 × 60	64 × 60 × 60	64	1 × 1	1	1
	Conv2D + BatchNorm2D	[[64 × 60 × 60] × 3 + Leaky ReLU] × 3	64 × 60 × 60	64	3 × 3	1	1
	AvgPool2D	64 × 60 × 60	64 × 30 × 30	64	3 × 3	2	1
	Conv2D + BatchNorm2D + Leaky ReLU	64 × 30 × 30	96 × 30 × 30	96	1 × 1	1	0
Block 1 skip connection	Conv2D + BatchNorm2D	32 × 60 × 60	96 × 30x × 30	96	1 × 1	2	0
Block 2	Conv2D + BatchNorm2D	[[96 × 30 × 30] × 3 + Leaky ReLU] × 3	96 × 30 × 30	96	3 × 3	1	1
	AvgPool2D	96 × 30 × 30	96 × 15 × 15	96	3 × 3	2	1
	Conv2D + BatchNorm2D + Leaky ReLU	96 × 15 × 15	288 × 15 × 15	96	1 × 1	1	0
Block 2 skip connection	Conv2D + BatchNorm2D	96 × 30 × 30	288 × 15 × 15	288	1 × 1	2	0
Block 3	Conv2D + BatchNorm2D	[[288 × 15 × 15] × 3 + Leaky ReLU] × 2	288 × 15 × 15	288	3 × 3	1	1
	Conv2D + BatchNorm2D	[288 × 15 × 15] × 2 + Leaky ReLU	288 × 15 × 15	288	3 × 3	1	1
	AvgPool2D	288 × 15 × 15	288 × 7 × 7	288	3 × 3	2	1
	Conv2D + BatchNorm2D + Leaky ReLU	288 × 7 × 7	512 × 7 × 7	512	3 × 3	1	1
Block 3 skip connection	Conv2D + BatchNorm2D	288 × 15 × 15	512 × 7 × 7	512	1 × 1	2	0
Average pooling	AdaptiveAvgPool2D (4 × 4)						
Dropout	(*p* = 0.5)						
Dense	Fully Connected	8192	150	150	1	–	–
Dropout	(*p* = 0.5)						
Batch normalization	Batch normalization 1D						
Dense	Fully Connected	150	2	2	1	–	–
Softmax							

In the above Eq. (15), W specifies the weight vector of network, U represents the uniform distribution that is drawn from range (-n, n) and z is the size of the previous layer.

#### Cross-domain adaptation of deep CNNs for mitosis classification

Deep CNNs have shown exemplary performance in diverse image classification tasks^9^. Deep networks learn features in a hierarchical manner, including generic and high-level features specific to the problem. This distinct characteristic allows us to reuse the pre-trained architectures for diverse tasks. Domain adaptation is a type of TL that allows reusing the already pre-trained architecture to a new problem. In this special type of TL, both target and source domains belong to different data distribution but are assigned to the same task. The advantage of this TL is gaining many low and intermediate level features that are shared among the diverse categories of images.

**Figure 9:**
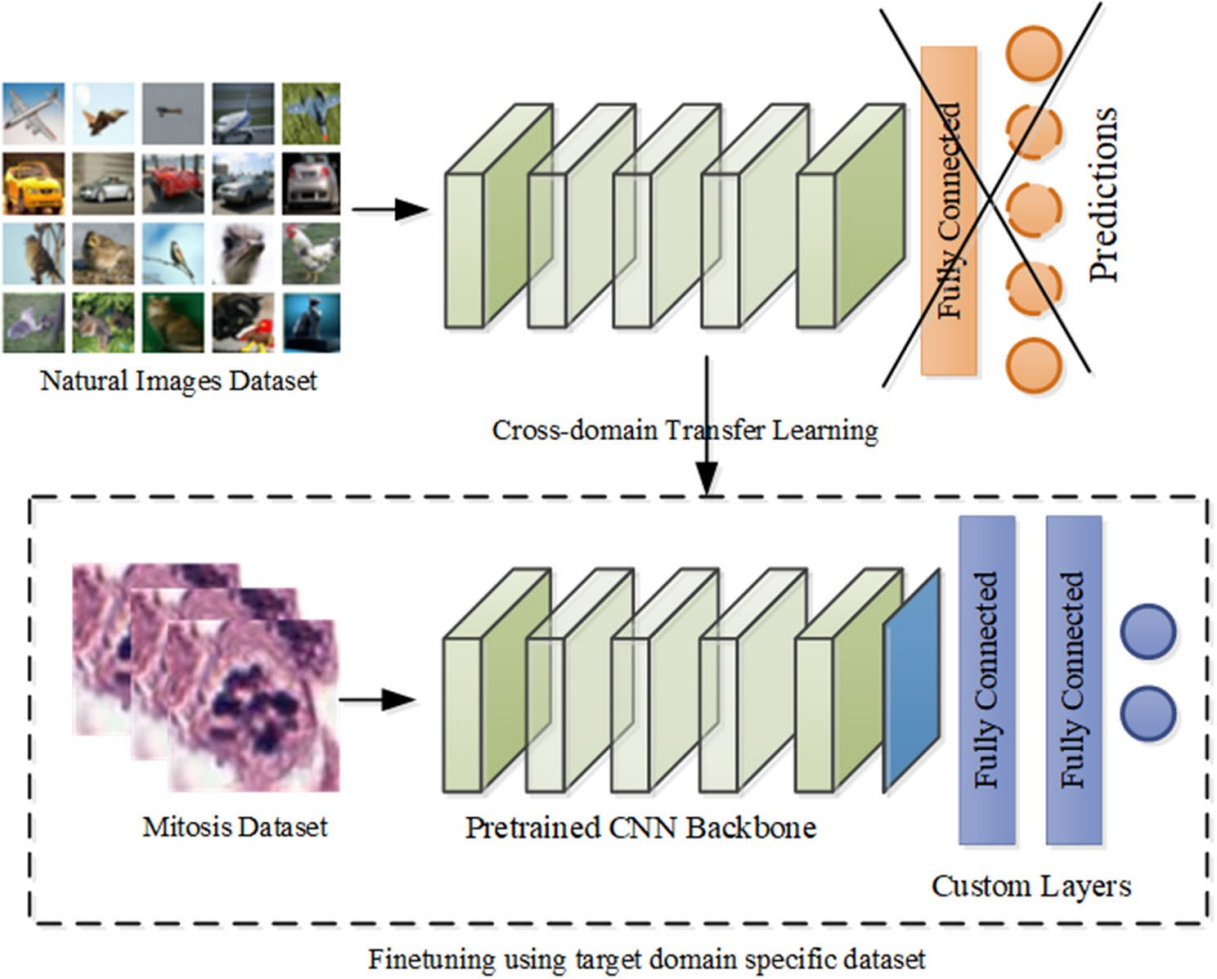
Cross-domain TL of the state-of-the-art CNN architectures.

In this study, we have exploited the concept of supervised cross-domain feature space adaptation and leveraged the knowledge of state-of-the-art deep CNNs models that are pre-trained on the ImageNet dataset for mitosis recognition problem. In this case, source $D(S) = \{ I_{N} ,P(I_{N} )\}$ and target $D(T) = \{ I_{H} ,P(I_{H} )\}$ domains share the low and intermediate level features ($I_{N} = I_{H}$) and both are assigned to the classification task but they follow different marginal distribution $P(I_{N} ) \ne P(I_{H} )$. Well-known deep CNN models: VGG, ResNet, DenseNet, ResNext, SENet and PolyNet with diverse architectural designs are modified by keeping feature extraction layers as such and adding average pooling layer in alliance with additional fully connected layers to align the network towards the mitosis specific features (shown in Fig. 9). The number of filters in task specific fully connected layers and filter size for adaptive average pooling are set based on the validation dataset (Table 2). Details of the architectures are mentioned in Table 4. The entire network is fine-tuned via the back-propagation algorithm on the mitosis dataset to obtain target domain specific semantically meaningful features.

**Table 4:** Cross domain adaptation of the state-of-the-art CNNs.

CNN backbone	Adaptive average pooling	Fully connect layers
SE-ResNet50	4 × 4	[4 × 4 × 2048, 950] [950, 2]
PolyNet-152	1 × 1	[2048, 2]
ResNext50	4 × 4	[4 × 4 × 2048, 750] [750, 2]
DenseNet201	7 × 7	[7 × 7 × 1920, 750] [750, 2]
VGG16	4 × 4	[4 × 4 × 512, 750] [750, 2]
ResNet18	4 × 4	[4 × 4 × 512, 750] [750, 2]

#### Mitosis classification using classical classifiers

Classical classification algorithms, including Naive Bayes, Logistic regression, SVM, Decision tree, Random Forest, and XGBoost, are also evaluated for mitosis detection problem. These classifiers were trained on the original feature space as well as on the HOG and LBP extracted feature space.

#### Performance metrics

The performance of the proposed Mitosis detection framework is evaluated using F-score, Recall, Precision, and Precision-Recall (PR) curve (Eqs. 16–18). Mitosis detection is a class imbalance problem, where under-representation of mitoses biases the performance measure that assigns equal weightage to each class. F-score evaluates the classifier’s correctness by computing the weighted average of precision and recall (Eq. 18). A recall is computed based on the criterion specified for automated mitosis detection modules^48^. According to it, mitosis in the telophase phase is under duplication; therefore, it is counted once, and all the mitoses that lie within 30 pixels of ground truth are counted as true mitoses.

16 $${\text{Recall }}=\frac{{{\text{Truely}}\,{\text{Predicted}}\,{\text{Mitoses}}}}{{{\text{Total}}\,{\text{Mitoses}}}}$$

17 $${\text{Precision }}=\frac{{{\text{Truely}}\,{\text{Predicted}}\,{\text{Mitoses}}}}{{{\text{Truely}}\,{\text{Predicted}}\,{\text{Mitoses}}\,{ + }\,{\text{Falsely}}\,{\text{Predicted}}\,{\text{Mitoses}}}}$$

18 $${\text{F {-} Score }}= 2 \times \frac{{{{{\rm Recall} \times {\rm Precison}}}}}{{{{\rm Recall} + {\rm Precison}}}}$$

In the above equation, recall suggests the detection rate, whereas precision shows the closeness of the predicted classes to the ground truth.

#### Data preparation for detection and classification models

Patches of size 512 × 512 pixels with 15% overlap were extracted for label refinement and mitosis detection at tissue-level stage for the training of Mask R-CNN. Whereas patches of 80 × 80 pixels were generated for the training of classification models. Input images for classification were resized to 120 × 120 and 224 × 224 pixels for custom and state-of-the-art CNN, respectively, using bilinear interpolation.

#### Parameter settings

Custom and pre-trained CNN architectures were fine-tuned using cross-entropy loss function by setting batch size equal to 16 and 6, respectively, assigning a learning rate of 1e−3, and weight decay of 1e−5 for 250 epochs. During training the model with best weight state was saved based on the validation dataset. Mask R-CNN was trained for 30,000 epochs using batch size 2, and the learning rate equal to 0.00025. The number of region proposals was set to be 128 for assigning mitosis and non-mitosis examples to ROI head for classification. Mask R-CNN and classification based CNN were optimized using SGD with warmuprestart and cosine annealing optimization strategy, respectively.

## Results

This study aims to develop a mitosis detection framework that can effectively learn the mitosis representation from the weakly labelled (centroid annotation) histopathological images. In this study, 61, 14, and 17 patients’ datasets (Table 2) were used for training, validation, and test, respectively. Mitosis detection is difficult due to its small size and close resemblance with non-mitotic nuclei. Therefore, we proposed a multi-phase detection approach, “MP-MitDet,” for mitotic nuclei discrimination. Multi-phase detection approach is split as: label-refiner and mitosis detection module. Mitosis detection module (Fig. 7) is further divided into: (1) mitotic region selection at tissue-level via deep instance-based detection and segmentation model, (2) blob analysis, and (3) mitotic region enhancement at cell-level via deep CNN classifier to establish the balance between detection rate and precision. Blob analysis is used as a post-processing step of detection module to prepare the input for cell-level classification phase.

The performance of the proposed detection module is evaluated for the maximum number of true mitoses against the small fraction of false positives (non-mitosis). Discrimination power is assessed on an independent test set, which was kept separate during the training and validation (Table 2). The generalization capacity is assessed by evaluating the proposed model on the test set with colour, contrast, and position variations.

### Label-refiner results

**Figure 10:**
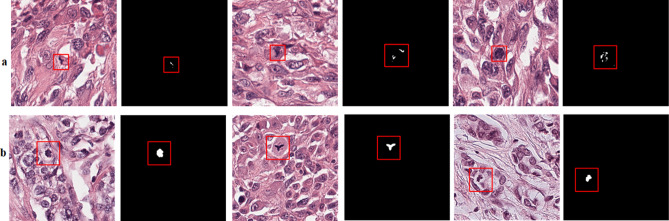
(**a**) Annotation results from blue-ratio binary thresholding (threshold value = 0.0075), panel (**b**) results of the proposed label-refiner that generates the pixel level image labels.

TUPAC16 dataset is provided only with centroid information of the mitotic nuclei, which is not sufficient for the training of supervised machine learning models, therefore, we used label-refiner to automatically annotate mitoses on tissue patches corresponding to 10 HFP area (results are shown in Fig. 10a). Mitosis annotation using blue-ratio binary thresholding produces imprecise annotation (Fig. 10b) because of shape irregularity and different configurations of mitosis in different phases of cell division (shown in Fig. 1). These labels are not sufficient for the effective training of the deep supervised detection model. Besides, manual annotation is not possible because of time-constraint. The performance of the supervised machine learning algorithms is dependent on the quality of the labels therefore, in label-refiner, we used Mask R-CNN to precisely define the morphology of the mitoses. We tried to avoid the loss of information by assigning semantic meaning via. circular annotator for those mitosis that were not segmented by the Mask R-CNN.

### Results of mitosis detection at tissue-level

**Figure 11:**
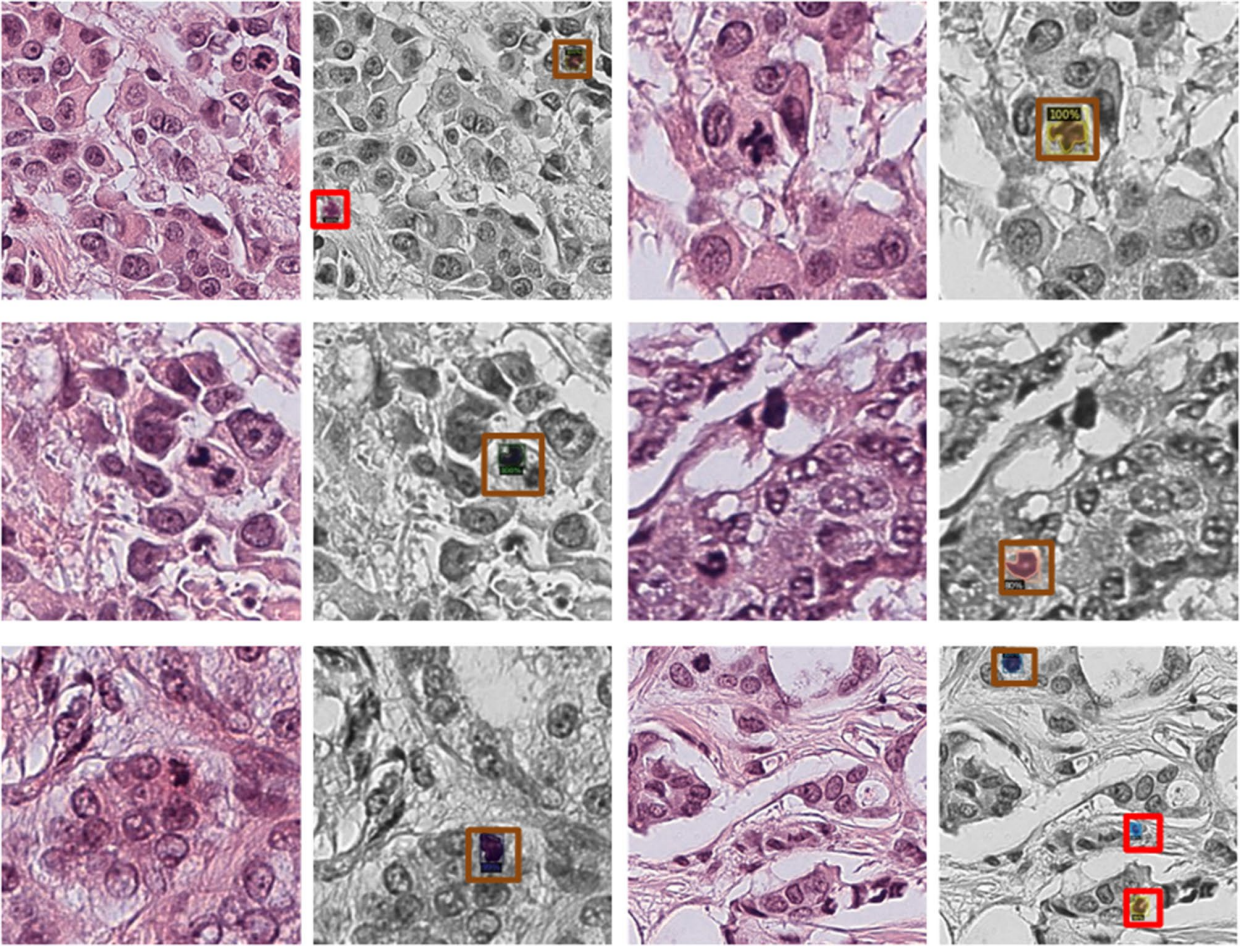
Results of the mitosis detection module at tissue-level, orange box highlights the true predictions whereas red box shows the false positive predictions (non-mitosis).

Mitosis detection is quite challenging due to small size, heterogeneous morphology, overlapping appearance, and their sparse representation. Relative abundance of non-mitotic nuclei undermines the performance of the detection model and makes it bias towards the class with large number of instances. The main challenge is to accurately identify the true mitotic nuclei with a minimum number of false positives. Therefore, initially, probable mitotic regions were isolated to reduce non-mitoses. For this purpose, detection module based on Mask R-CNN was fine-tuned on the training dataset with refined labels so that the maximum number of mitoses were retained with a minimum false positive rate. The results of the detection model on the test dataset are shown in Fig. 11. The distinct feature of Mask R-CNN is that it initially screens the candidate regions; subsequently, detection and segmentation algorithm is applied only to selected regions to distinguish the mitosis from other cellular components. The advantage of detection of candidate mitotic regions at tissue level is to reduce the class imbalance between mitosis vs. non-mitosis examples while persisting hard examples that mimic the mitosis representation. This results in relatively balanced dataset with loss of only two mitoses on test set (results are shown in Table 5).

**Table 5:** Results of mitosis detection at tissue-level.

Detection model	Precision	Recall
Proposed Mitosis detection module	0.1	0.98
Wahab et al.*^37^	0.07	0.92

### Results of mitosis classification at cell-level

In the proposed detection technique, after getting probable mitotic regions from the detection model, the output is assigned to a classifier to enhance the final detection by reducing the false positives. For the refinement of detection results, different classifiers are evaluated that are discussed below. The classifier’s performance is assessed using an F-score, which is a criterion suggested by all mitosis detection challenges because of the imbalanced nature of the dataset. Moreover, true positive detection rate and precision are also considered to evaluate the overall performance. The PR curve is used for the evaluation of the discrimination power of the model at different threshold values.

### Mitosis classification using conventional classifiers

Initially, the potential of conventional machine learning algorithms such as SVM, Logistic Regression, Decision Tree, Naïve Bayes, Random Forest, and XGboost were evaluated for the discrimination of mitotic nuclei. These classifiers were trained and validated on probable mitotic cells of train and validation sets (Table 2), respectively that were generated from detection model (Fig. 7b). The performance analysis on test images in terms of F-score suggests that conventional classifiers do not have enough representation ability to discriminate between two classes (results are shown in Table 6). The probable reason for poor performance was the complex nature of data; moreover, such classifiers’ performance is highly dependent upon the feature space of training data.

**Table 6:** Performance analysis of classical classifiers on test dataset.

Classification model	F-score ± S.E	Precision	Recall	F-score ± S.E	Precision	Recall	F-score ± S.E	Precision	Recall
	RGB			LBP			HOG		
SVM	0.21 ± 0.023	0.14	0.37	0.26 ± 0.025	0.35	0.20	0.18 ± 0.022	0.13	0.28
Logistic Regression	0.15 ± 0.020	0.10	0.33	0.1 ± 0.017	0.1	0.12	0.17 ± 0.021	0.39	0.10
Naïve bayes	0.12 ± 0.018	0.09	0.23	0.18 ± 0.022	0.44	0.11	0.14 ± 0.020	0.38	0.087
Decision Tree	0.19 ± 0.022	0.13	0.40	0.16 ± 0.021	0.42	0.10	0.14 ± 0.020	0.379	0.09
Random Forest	0.18 ± 0.022	0.25	0.14	0.19 ± 0.022	0.15	0.25	0.05 ± 0.012	0.03	0.11
XGBoost	0.34 ± 0.027	0.29	0.40	0.22 ± 0.024	0.15	0.39	0.10 ± 0.017	0.08	0.12

### Mitosis classification using state-of-the-art deep CNNs

In literature, several state-of-the-art deep CNN architectures are proposed that have shown convincing performance on a very large image dataset, ImageNet^34,35,45,49,50^. In this work, we have evaluated the potential of these architectures by adding additional layers and fine-tuned them on the mitosis dataset using the backpropagation algorithm. The results of state-of-the-art deep CNNs: VGG, ResNet, DenseNet, ResNext, SENet and PolyNet are mentioned in Table 7.

**Table 7:** Performance of the state-of-the-art CNN adapted via cross-domain TL on test dataset.

Classification model	F-score ± S.E	Precision	Recall
Mit-VGG	0.67 ± 0.027	0.61	0.74
Mit-ResNet	0.71 ± 0.026	0.66	0.77
Mit-DenseNet	0.72 ± 0.026	0.65	0.82
Mit-ResNext	0.72 ± 0.026	0.68	0.75
Mit-SeNet	0.66 ± 0.027	0.64	0.69
Mit-PolyNet	0.65 ± 0.027	0.58	0.74

### Mitosis classification using the proposed deep MitosRes-CNN

**Figure 12:**
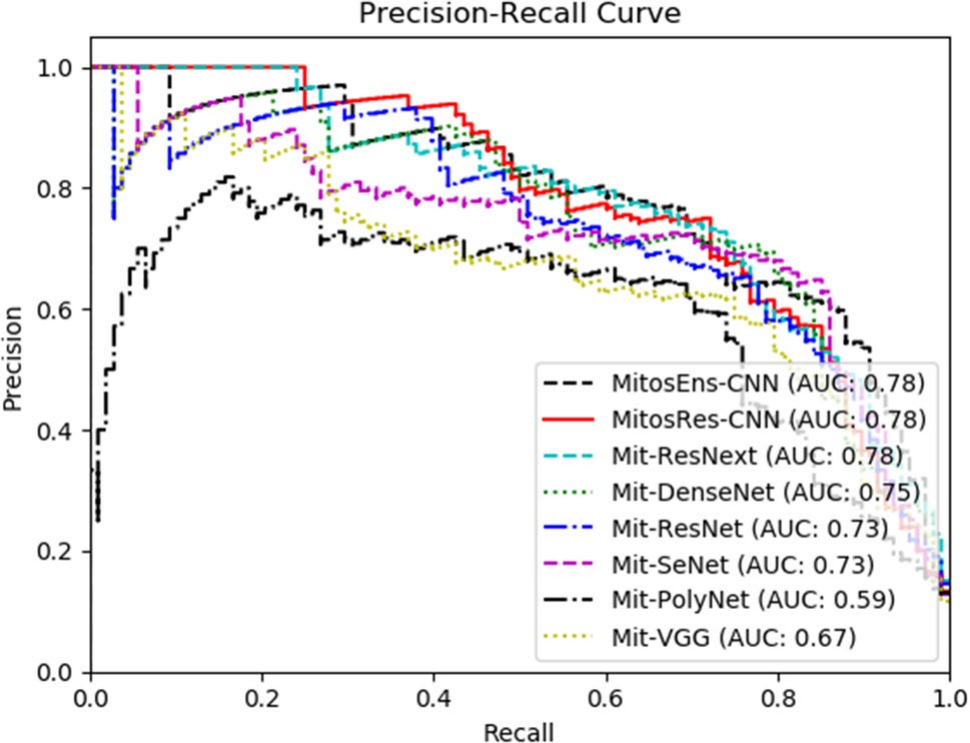
Discrimination analysis via PR curve.

Proposed deep “MitosRes-CNN” is trained from scratch on the mitosis dataset using the backpropagation algorithm, and it was evaluated on test images (Table 2). The results of deep CNN are shown in Table 8 and Fig. 12. The performance comparison of the proposed “MitosRes-CNN” with state-of-the-art CNNs (Tables 7 & 8) suggests that the proposed model is good in discrimination (F-score: 0.73) of mitoses with significant precision (0.70), recall (0.76), and AUC of PR curve (0.78). The primary objective is that the proposed model “MitosRes-CNN” shows good generalization when applied to different variations of datasets. Therefore, generalization of the proposed detection model is evaluated by assigning a dataset generated through semantic segmentation^29^ that was not made part of the training set. Moreover, the performance of the proposed classifier is evaluated on position variant, colour, and contrast variant examples (Table 9). The significant detection rate with adequate precision (Table 9) on various datasets suggests that proposed deep “MitosRes-CNN” has invariant feature learning ability and is robust against variations.

**Table 8:** Performance comparison of the proposed model with existing techniques on test dataset of TUPAC16 auxiliary dataset, whereas (*) shows same patient distribution in the test set.

Classification Model	Model type	F-score ± S.E	Precision	Recall
Proposed MitosEns-CNN*	CNN based ensemble	0.75 ± 0.025	0.734	0.768
Proposed MitosRes-CNN*	CNN	0.73 ± 0.025	0.70	0.76

Existing Techniques	Model Type	F-score	Precision	Recall
Wahab et al.*^37^	CNN based ensemble	0.713	0.77	0.66
Li et al.^15^	CNN	0.717	–	–
Paeng et al.^30^	CNN based ensemble	0.73	–	–
Akram et al.^36^	–	0.65	0.68	0.62
Mahmood et al.^51^	CNN based ensemble	0.64	–	–

**Table 9:** Robustness of the proposed MitosRes-CNN on various datasets.

Variation type	Detection rate	Precision
Position variation	0.83	0.66
Contrast variation	0.70	0.69
Brightness variation	0.70	0.66
Saturation variation	0.72	0.67
Dataset from Wahab et al.^29^	0.73	0.62

### Mitosis classification via consensus of multiple classifiers

For reliable analysis of histopathological images, usually, a decision is taken from more than one pathologist. Moreover, most of the proposed mitosis detection models in the literature are ensemble-based. To emulate the pathological process, we proposed score level fusion from multiple classifiers. Therefore, each sample is assigned a class by taking a confidence score from three different classifiers: MitosRes-CNN, Mit-DenseNet, and Mit-ResNext and a final decision is made based on the highest confidence score. The proposed ensemble of CNNs “MitosEns-CNN” results in improved performance (Table 8) in terms of F-score (0.75), precision (0.73), recall (0.768), and discrimination ability (AUC: 0.78).

## Discussion

In this work, we have developed a deep CNN based multi-phase mitosis detection framework “MP-MitDet” for Breast cancer histopathological images. The automation of histopathology based analysis of mitotic nuclei can lessen the workload of pathologists and time cost of manual count.

In this work, we have proposed a new labelling scheme to generate pixel-level annotations for centroid annotated images. The proposed label-refiner combines precise morphological information with semi-semantic information. This annotation scheme overcomes the downsides of thresholding based technique used by different researchers^37^, which resulted in many disconnected false-positive blobs and overlooked true positives. Likewise, circular labelling scheme^15,16,53^ is unable to provide complete morphological information. In our work, the label-refiner improves the labels by exploiting the prediction maps generated by deep instance based segmentation model Mask R-CNN. Whereas, the mitoses that are missed by the Mask R-CNN are represented via pseudo labels to assign them semantic meaning in the form of circular annotations. This thus results in a combination of both fine-grained information gathered from exact morphology learned via Mask R-CNN and coarse-level information incorporated from circular labels. The addition of pseudo-labels in alliance with explicit mitotic representation adds randomness, which improves the generalization of a deep learning model by preventing them from memorizing specific shape.

We have performed the mitosis detection on histopathological images by discriminating both on tissue and cell-level. The main bottleneck in the development of an automated detection system is intra-class heterogeneity^4,54^ and class imbalance. The analysis at two different levels addresses the challenge of accurately identifying mitoses with minimum number of false negatives while reducing class imbalance. The first stage that determines the probable mitotic regions by analysing the 10 HPF tissue region excludes the undesired tissue stoma and a large proportion of non-mitotic nuclei and other organelles similar to mitosis^5^.

The significance of employing Mask-RCNN at firsts stage is that it is an instance based segmentation and detection approach, which marks each mitosis separately from the others. The instance level analysis makes Mask R-CNN more appropriate for this problem compared to semantic segmentation. Semantic segmentation performs pixel-wise classification and treats each mitosis as a group of objects. Moreover, the output of semantic segmentation requires post-processing to categorize set of clustered objects as single mitosis and distinguish each mitosis from one another.

In the second stage of detection, a classifier is used to categorize the selected cells as either mitosis or non-mitosis. The advantage of this stage is that it reduces the false positives and improves the precision by learning the characteristic features of mitosis and hard-to-classify negative examples. We have proposed a simple yet deep CNN “MitosRes-CNN” for the classification. As selected cells are small in size (80 × 80 pixels) and complex in the pattern, therefore, a complex architecture with a large number of filters can lead to overfitting on the training dataset. The exploitation of multiple transformation in a simple way with the idea of the effective receptive field and residual learning in the proposed CNN helps in learning abstractions at different levels while maintaining good generalization. Additionally, the comparison of the proposed MitosRes-CNN with state-of-the-art CNN architectures (Tables 7 and 8) suggests that it was possible to positively train the custom deep CNN on a moderately-sized training set of 61 patients without transfer learning.

This study also demonstrated that the state-of-the-art CNN architectures designed for non-medical tasks could be effectively adapted for mitosis detection by adding task-specific layers, selecting appropriate hyperparameters, and finetuning them via cross-domain TL. We have further shown that the exploitation of the learning experiences of different deep CNN architectures through decision score based ensemble can result in the significant gain in F-score, precision, and recall.

The multi-phase detection framework proposed in this work is simple in design and different from approaches reported in literature^28,30^ who also performed detection at two levels. The aforementioned approaches passed the segmented output to the classifier in the first phase to identify the hard negative examples. In the second step, easy-to-classify negatives examples are undersampled in the training data, while hard examples are augmented. Contrary to this, in our proposed “MP-MitDet” approach, we have performed cell-level classification for output of the detection phase without additionally mining the hard instances. The promising performance of the proposed “MP-MitDet” approach on the TUPAC16 dataset in terms of F-score, recall, and precision compared with the existing techniques (Table 8) and on various combinations of datasets (Table 9) suggests good generalization ability. The proposed approach has still a margin of improvement. In this study, a simple score fusion-based ensemble approach is targeted; however, the potential of advance ensemble learning techniques will be explored in the future. Moreover, the learning capacity of different CNNs can be exploited by combining feature spaces of diverse CNN architectures and assigning their output to meta-classifier for final decision.

## Conclusions

According to the Nottingham grading scheme, the mitosis count is the fundamental prerequisite of breast cancer tumour grading and proliferation rate. In this work, we proposed a deep multi-phase mitosis detection framework, “MP-MitDet,” for mitosis discrimination on H&E stained breast tissue histology images. The promising performance of “MP-MitDet” in terms of F-score of 0.73 and 0.75 for custom and ensemble-based CNNs, respectively suggests the effectiveness of solving complex problem at multiple levels. We have shown that the refinement of weakly annotated labels with semantic level labels and segregation of numerous non-mitotic nuclei at tissue level with Mask R-CNN helps in attaining high mitosis detection rate (0.98) with low density of false mitosis examples. The challenges of heterogeneous mitotic morphologies and inter-class resemblance are resolved by developing custom CNN “MitosRes-CNN” that enhance the results by significantly reducing mitosis-like-hard example with a significant precision (0.70 for custom, 0.73 for ensemble based CNN) on the test dataset. Promising performance of the proposed “MP-MitDet” on different variations of dataset and its comparison with existing techniques suggests its good generalization and potential to learn characteristic features of mitosis. In future, it can be adapted to reduce the decision space of the pathologist by suggesting the probable mitotic regions.

## Data Availability

All the datasets used in this work are publicly available, whereas datasets that are generated or analysed during labeling, detection and classification are available from the corresponding author on reasonable request.
